# Reclaiming microbiology: scientists as community members and advocacy leaders

**DOI:** 10.1099/mgen.0.001533

**Published:** 2025-10-15

**Authors:** I’ah Donovan-Banfield, Nikea Pittman, Ariangela Kozik, Kishana Taylor, Chelsey Spriggs, Eva Davis

**Affiliations:** 1 Black Microbiologists Association, Detroit, MI, USA; 2 Department of Biochemistry, Cell & Systems Biology, Institute of Systems, Molecular and Integrative Biology, University of Liverpool, Liverpool, UK

**Keywords:** community engagement, diversity, equity, inclusion, inclusivity, marginalized scientists, public health

## Reclaiming microbial science

The Black Microbiologists Association (BMA) is a self-assembled community of Black microbiologists that formally registered as a non-profit organization in 2021 [1]. Black in Microbiology Week, our flagship event, is a living embodiment of BMA’s core values. This virtual conference was deliberately designed for free and global access, dismantling traditional barriers to scientific engagement and knowledge sharing [2]. For microbial scientists, whose work depends on public trust, health equity and global collaboration, the impact of our work extends beyond laboratories.

At its heart, Black in Microbiology Week is powered by community-led programming, meaning we endeavour to offer content that directly addresses the authentic needs and interests of the microbiologists in our membership. Its interdisciplinary reach within the broader field of microbiology (e.g. across bacteriology, virology, mycology and parasitology, host–microbe interactions and infectious disease public health) ensures that the complexity of microbiology is represented from various perspectives, fostering rich discussions and collaborations across sub-disciplines. We intentionally spotlight early-career researchers, providing them with crucial visibility and a platform to share their research with a global audience. We also strive to integrate discussions on policy, community engagement and advocacy, bridging the gap between scientific discovery and broader societal impact. Black In Microbiology is an opportunity for us to showcase our commitment to sustained community care, empowering our members to support and uplift their own communities and ensure that their science reaches beyond the lab. In this article, we will explore what can be done, both as a self-organized community of historically marginalized microbiologists and as a scientific community as a whole, to reclaim the narrative surrounding microbial sciences going forward.

## Scientists as community members

BMA highlights advocacy, visibility and community engagement as integral to the practice of being a scientist. This is because *scientists are connected to our community*. We commonly involve local communities in our research, deliver workshops to the public and contribute to news articles, podcasts or other forms of civic engagement. Importantly, *scientists are products of our community*. Our career values are informed by each person’s cultural heritage, educational experience and personal history. In fact, when science classrooms integrate social topics, this strengthens student learning [3]. As a discipline, *scientists are constantly contributing to their community*. By applying systemic methods to investigate difficult problems, this leads to innovation. Today, we argue that challenges in equity and inclusion should be addressed with the same rigour. We need to use targeted strategies to remove barriers excluding Black and other marginalized scientists from science careers and education [4,7].

## Infrastructure for inclusion: a case study on Black In Micro

Globally, microbiologists are seeking to increase a sense of belonging for everyone [8,12]. As a result, society has witnessed a new form of advocacy that is rooted in social media [13,17]. This article highlights the impact of BMA on the Science, Technology, Engineering and Mathematics (STEM) community. ‘Black In Micro’ scientists and supporters have worked ceaselessly to amplify the voices of marginalized scientists. In 2021, BMA became a non-profit organization to secure the ability to accept donations and fund new initiatives. Our public-facing website is designed as a resource for everyone seeking community and those looking to expand their networks and strengthen hiring practices (www.blackinmicrobiology.org).

Sustainability requires not only events but also systems of resourcing, mentorship and visibility. To address the exclusion of Black microbiologists, we launched our flagship conference, Black in Microbiology Week, in September 2020. This event was a celebration of the contributions of Black microbiologists past and present. Since then, we have delivered two further Black in Microbiology Week events, platforming over 100 Black scientists from across the world. Through these events, we have engaged over 4,000 participants from more than 80 countries to spearhead conversations around the future of STEM education and public perception of microbiology as a field and showcased the accomplishments of early-career microbiologists (Table 1).

**Table 1.** Strategic initiatives driven by BMA

Collective experiences of Black scientists across social media	Response from Black In Micro	Impact on the STEM community
Disconnected from welcoming communities of support, with lack of visibility in STEM disciplines	Launched Black In Microbiology Week, a recurring online celebration	More than 4,300 registered participants representing 80+ countries, 57% early career and 66% women and gender minorities Established #BlackInMicro hashtag to help people find Black scientists online and grew to a following of 10,000+ on social media
Less likely to have their impact acknowledged, featured and cited	Developed widely attended events and new partnerships with media outlets	Over 100 Black speakers featured during Black In Micro Week BMA and its members featured in 40+ articles, podcasts and interviews
Smaller social networks to receive informal mentorship, collaborate or share cultural wealth	Created year-round opportunities for networking	Free membership with access to online discussion board and webinars Arranged events for members travelling to the same conferences to meet in person
Excluded from learning about professional opportunities and skills to progress career advancement	Provided tools for job searches and career growth	Created a job board featuring postings across multiple sectors Past events have focused on resume building, grant writing and tips to secure grad school, postdoc or professional roles
Fewer invitations to be featured in articles, interviews or join collaborations	BMA acts as a point of contact for external groups and shares information with members	Newsletter established to advertise opportunities internally Created BMA Talent Network to feature 80+ Black microbiologists; expanding and relaunching by 2026

## Harmony in the face of uncertainty: Black In Micro Week 2025

In 2025, we are celebrating 5 years of Black In Microbiology. Since its inception, we have prioritized highlighting the impact made by Black scientists, meeting the needs of early-career scientists and strengthening career success. By meeting online, we bring together Black scientists from across the diaspora, fostering a global community. This year, Black In Micro Week takes place October 14–16, which coincides with UK Black History Month. The conference theme is centred on HARMONY: *H*ope, *A*lliance, *R*esilience, *M*utual support, *O*ptimism, *N*urturing and *Y*ielding progress. Through these concepts, we emphasize community harmonization as resistance to current and future challenges.

**Fig. 1.**
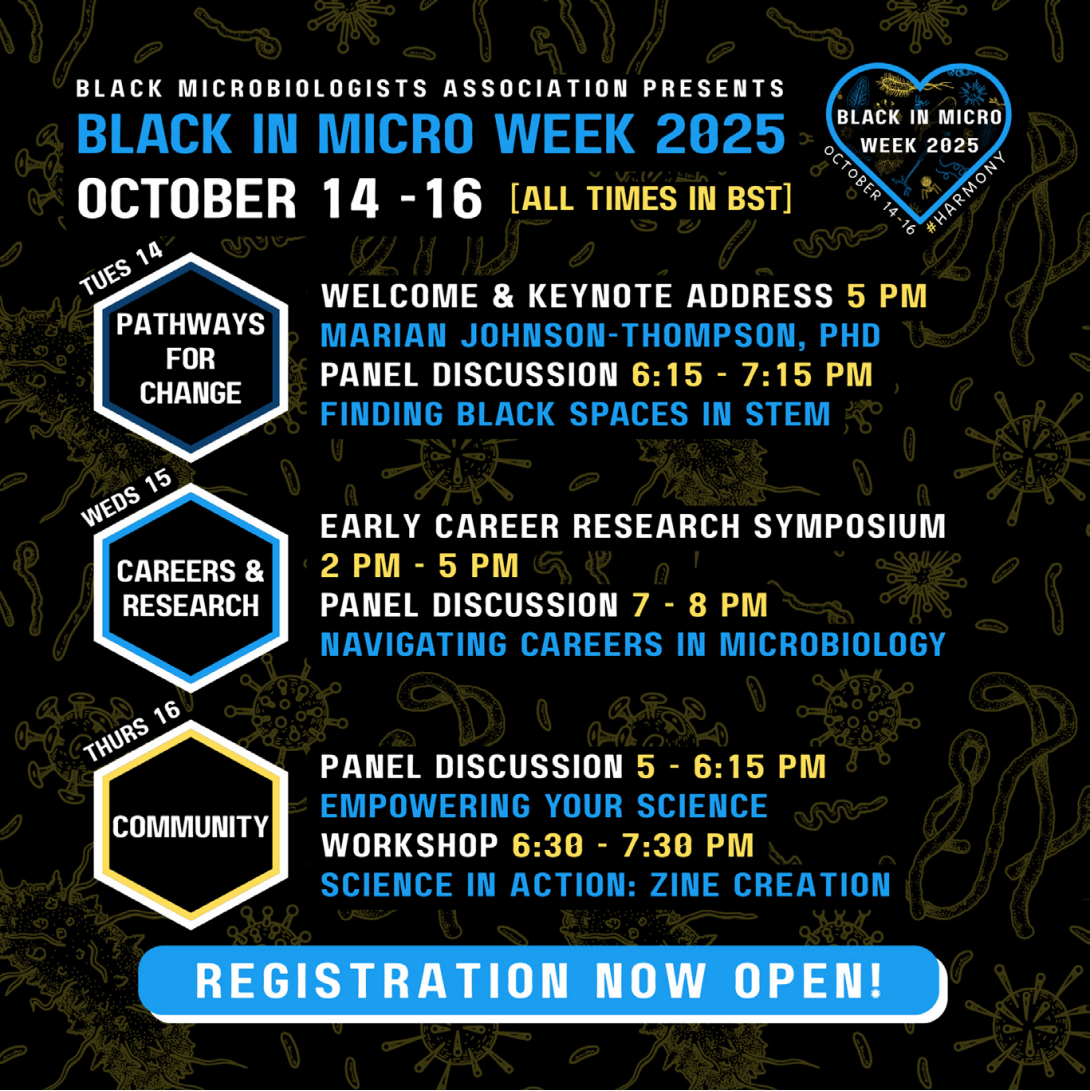
Programme overview for Black in Microbiology Week 2025. Event times are given in British Summer Time (GMT+1).

The conference kicks off with a day focused on *Pathways for Change* (Fig. 1). The keynote speaker, Dr Marian Johnson-Thompson, will discuss key milestones in the history of advocacy among microbiologists and what history tells us about the importance of community in microbiology. This will be followed by a panel discussion on ‘Finding Black Spaces in STEM’, which will draw perspectives on how we can use the lessons of the past to build a more prosperous and fair future for Black scientists. On *Careers and Research* Day, the Early Career Research Symposium features 25 microbiologists and their research from across the world. The ‘Navigating Careers in Microbiology’ panel presents scientists who have explored careers outside of the academic pipeline. Black In Micro Week concludes with a spotlight on *Community*, where panellists in ‘Empowering your Science’ will discuss how to ethically engage communities for sustainable collaborations. The final session includes a zine-making science communication workshop and networking opportunities, where our attendees can start to mobilize on initiatives that will help to increase equity and harmony across microbiology.

As scientists navigate increasing challenges and scrutiny, the need for robust, supportive communities like ours has never been more urgent. This is more than just an event; it’s a vital hub for connection, empowerment and advocacy. We are proud to provide closed captioning and American Sign Language interpretation at all events to ensure that our programming is accessible to as many as possible. Due to our commitment to reduce barriers to attendance, Black in Microbiology Week is offered at no cost to attendees. However, we welcome and truly appreciate any donations, large or small. Any generosity directly supports our initiatives and helps to ensure we can continue to provide free, accessible programming for our community. Join us in making a tangible difference. Registration and donation information can be found here. We hope to see you there.

## Legacy and the invitation to join

The future of BMA, at this critical juncture, is one of deliberate expansion, not exception or exclusion. Our journey has shown that the scientific community flourishes when every voice is valued and every contribution is celebrated. Black in Microbiology Week 2025 will further amplify this expansive vision, highlighting the importance of recognizing that science is critical to society and that scientists are members of the broader community. Our collective experience underscores that the pursuit of equity and excellence in science is a shared responsibility that requires the unwavering commitment of all who believe in a more just, innovative and robust future. We affirm that this work, far from being ‘discriminatory’, is an essential corrective to the historical, repeated, documented exclusion of certain groups of people from microbiology and science more broadly. We extend an urgent and open invitation to funders, institutions and individuals alike. Your partnership is essential in this effort. The work towards a scientific enterprise that works for all, and that benefits the greater good, must be intentional, adequately resourced and sustained over the long term. Through these efforts, it is our hope that we can transform the scientific landscape, ensuring its benefits are both universally accessible and universally felt.

## Abbreviations

BMA: Black Microbiologists Association
